# Cellulose Membranes in the Treatment of Spent Deep Eutectic Solvent Used in the Recovery of Lignin from Lignocellulosic Biomass

**DOI:** 10.3390/membranes12010086

**Published:** 2022-01-13

**Authors:** Vadim Ippolitov, Ikenna Anugwom, Robin van Deun, Mika Mänttäri, Mari Kallioinen-Mänttäri

**Affiliations:** 1 Department of Separation Science, LUT School of Engineering Science, LUT University, P.O. Box 20, 53851 Lappeenranta, Finland; Ikenna.Anugwom@lut.fi (I.A.); Mika.Manttari@lut.fi (M.M.); Mari.Kallioinen-Manttari@lut.fi (M.K.-M.); 2 Department of Applied Engineering, Chemical Engineering Technology, University of Antwerp, 2610 Wilrijk, Belgium; robin.vandeun@student.uantwerpen.be

**Keywords:** DES, ultrafiltration, biomass treatment, concentrating, solvent purification, lignin recovery, cellulose membrane

## Abstract

Ultrafiltration was employed in the purification of spent Deep Eutectic Solvent (DES, a mixture of choline chloride and lactic acid, 1:10, respectively) used in the extraction of lignin from lignocellulosic biomass. The aim of this was to recover different lignin fractions and to purify spent solvent. The results revealed that the commercial regenerated cellulose membranes—RC70PP and Ultracel 5 kDa UF membranes—could be used in the treatment of the spent DES. The addition of cosolvent (ethanol) to the spent DES decreased solvent’s viscosity, which enabled filtration. With two-pass ultrafiltration process with 10 kDa and 5 kDa membranes about 95% of the dissolved polymeric compounds (lignin and hemicelluloses) were removed from the spent DES. The utilized membranes also showed the capability to fractionate polymeric compounds into two fractions—above and under 10,000 Da. Moreover, the 10 kDa cellulose-based membrane showed good stability during a continuous period of three weeks exposure to the solution of DES and ethanol. Its pure water permeability decreased only by 3%. The results presented here demonstrate the possibility to utilize cellulose membranes in the treatment of spent DES to purify the solvent and recover the interesting compounds.

## 1. Introduction

Deep eutectic solvents (DESs) are mixtures of compounds with a melting point significantly lower (>50 °C) than the melting points of each of the individual compounds [1,2]. The melting point depression occurs due to the shift of the electric charge between a hydrogen bond acceptor (HBA) and a hydrogen bond donor (HBD). This delocalization moves from an ion, which is the HBA, to a hydrogen-donor moiety, which is the HBD, along with the hydrogen bonding between them. Usually, the HBA is represented by a quaternary ammonium salt, while the HBD is often represented by amides, carboxylic acids, and alcohols [3].

Since the first description of DESs by Abbott et al. [1], they have been studied as “green solvents” for various applications. Due to their unique physical and chemical properties, such as thermal stability, nonvolatility, and nontoxicity [4], DESs pose a significant interest. They are also easy to prepare, and they are generally biodegradable [5,6]. Owing to the unique properties of DESs, they have been successfully applied as solvents from electrochemistry to the extraction of organic compounds (hydrocarbons and oils), metal species, and bioactive materials [3,7]. DESs, including mixtures of lactic acid and choline chloride, are appropriate solvents for biomass delignification [8]. The DES-based biomass fractionation process has been studied for the possibility of it being more effective in terms of lignin extraction compared to the traditional kraft or organosolv processes [9].

Lignocellulosic biomass has a complex structure and high recalcitrance [10]. The cross-linked recalcitrant structure of lignocellulose biomass poses a difficulty for its fractionation. Lignin has a very complex structure, and it is difficult to dissolve from lignocellulosic biomass [11]. DESs have been actively studied as solvents for lignocellulosic biomass treatment [12,13,14,15,16] because they demonstrate dissociation abilities towards aryl ether bonds and carbon-carbon bonds as well as towards lignin-carbohydrate complex [17].

As “green solvents,” DESs can be a suitable candidate to substitute ionic liquids (ILs) due to the better performance of DESs in terms of operational cost and environmentally friendliness, which here means, for instance, the minimal toxicity towards higher organisms [7]. The greatest limitation that hinders the introduction of IL-based processes to the industry is their considerably high cost, which is related to expensive preparation, and ineffective recycling [18]. Even though the preparation of DESs is easier and cheaper compared to that of ILs, ineffective recycling also limits their utilization as solvents. In spite of the fact that the use of DESs in different applications has been widely studied, the investigation of DES regeneration and recycling is still in its infancy.

The requirements for the recycling process for DESs depend naturally on the application. When a DES is used to dissolve lignin from biomass, the lignin can be recovered from the DES via precipitation with water [19]. Briefly, anti-solvent addition causes the solubilized solids to precipitate from the solvent. Precipitates are removed with solid-liquid separation. Eventually, the anti-solvent can be evaporated. The remaining liquid is DES that can be further recycled. However, impurities such as sugars derived from hemicellulose hydrolysis and phenolic compounds are left in the DES, and they should be removed [20]. Furthermore, the recycling process should produce a purified DES, in which the ratio of its components is close to their ratio in the original DES, because the ratio of the components might have a significant influence on the efficiency of the DES as a solvent.

Membrane filtration is an attractive technology for DES treatment to enable its recycling, as it is possible to simultaneously purify and concentrate it in a membrane process in the same process step. Furthermore, the separation efficiency is easy to tailor via membrane choice. In addition, membrane processes are modular and easily scalable processes. As well as this, it is beneficial that no phase change occurs while using the pressure-driven membrane filtration processes. Despite the attraction of using a membrane process in the recycling of DESs, there is only a very limited number of reported studies on this issue.

Pressure-driven membrane filtration of spent DES as a purification method has not been studied intensively [21,22].

Liang et al. [21] reported a combination of ultrafiltration (UF) and electrodialysis (ED) with the aim of separating a DES into its constituents (choline chloride and ethylene glycol) for further use. In their study, Liang et al. used UF membranes made from polyethersulfone (PES), the aim of which was to recover lignin degradation products from the spent DES to enhance the recycling of the DES as the permeate of the UF process or to enable the efficient use of the ED process for DES purification. The authors reported that the combination of the two membrane processes (UF + ED) led to a recycled DES with an 8.7% loss in lignin separation efficiency compared to the original DES efficiency. The purification of the spent DES only with the UF membrane (0.65 kDa) resulted in a 25.5% loss in lignin separation efficiency. The authors did not present any values to describe membrane performance, such as flux or permeability. They filtered DES, which was diluted with the antisolvent (water), which might have been facilitating the membrane filtration. As such, a DES has a typically high viscosity, which limits efficient membrane filtration.

Some DESs such as Choline Chloride: Lactic acid (molar ratio 1:10) demonstrate extremely poor ability to dissolve cellulose [8,23]. Thus, in this study, the possibility to use commercial cellulose membranes for the treatment of spent DES was evaluated. The idea was to use ultrafiltration to produce concentrated fractions, from which lignin could be recovered via precipitation with the antisolvent (water). This approach originates from the need to minimize water consumption and simplify the purification process required for the recycling of a DES. In an ideal case, lignin could be recovered to the concentrate fraction of the ultrafiltration step, and the permeate produced with the UF membrane would be a purified DES for reuse.

## 2. Materials and Methods

### 2.1. Materials

For the materials of this study, air-dry birch (*Betula pendula*) wood was provided by a local wood mill. In addition, the commercially sized chips (typically 25–35 × 0–25 × 2.5–6 mm, L × W × T) were milled to the size of 1 mm using a hammer mill. The lignin content was determined by acid hydrolysis using the standard NREL method. Lactic acid (LA, 90% purity), ethanol (ETAX A, 99.9% purity), and technical acetone (99.5% purity) were acquired from VWR (Radnor, PA, USA), while choline chloride (ChCl, 99% purity) was supplied by Acros Organics (Geel, Belgium). Ethanol (ETAX B, 92% purity) and sodium chloride (>99.5% purity) were purchased from Altia Industrial (Colorado Springs, CO, USA) and Sigma-Aldrich (St. Louis, MO, USA), respectively. Throughout this work, ultra-pure deionized water (DI, 15 MΩ, 0.5–1 μS/cm), which was produced with CENTRA-R 60\120 system (Elga purification system, Veolia Water, UK) and is referred to as DI water in this work, was used for washing and as an anti-solvent in all the preparations of the solutions.

Ultrafiltration was performed with the RC70PP membrane (Alfa Laval company, Lund, Sweden) and the Ultracel UF Discs 5 kDa (Millipore company, Burlington, MA, USA). Table 1 presents information on the technical characteristics of the membranes used in the experiments, which is based on data provided by the manufacturers.

#### 2.1.1. DES Preparation

The DES used in this study was prepared according to the procedure described by Esmaeili et al. [24] with a slight modification in the molar ratio from 1:9 to 1:10 ChCl:LA. The modification in the molar ratio was made due to several studies, which reported that a decrease in the amount of choline chloride led to an increase in lignin removal from the biomass [25,26,27]. Briefly, a mixture of ChCl and LA with the molar ratio of 1:10 was vigorously stirred for 2 h at 80 °C, until a transparent and homogeneous mixture was obtained. This obtained DES solution was then transferred into a glass container, which was then sealed, where it was stored for further use.

#### 2.1.2. Biomass Treatment

A moisture-free milled birch fraction (~2 × 2 *×* 0.1 mm) was used in the DES treatment, which was conducted by mixing birch wood and DES with the liquid to a solid ratio of 10:1 g/g. The treatment was performed with constant stirring at 120 °C for 4 h. After the experiment, the spent DES was separated from the solid fraction by vacuum filtration with a Buchner funnel. The residue was then washed with the mixture of ethanol (92% purity) and DI of a volume ratio of 2:1. Subsequently, the treated biomass was washed for the second time by acetone.

### 2.2. Dilution of the Spent DES to Decrease Viscosity

The spent DES as such has a high viscosity (154.35 mPa × s), which limits the efficient membrane filtration. One solution to this problem is the dilution of the spent DES by the addition of a cosolvent. However, the cosolvent should not cause abundant lignin precipitation, and it should not interact chemically with the membrane material. In this study, ethanol (99%) was chosen to be used as a cosolvent. To evaluate the influence of ethanol addition to the viscosity of the spent DES, 50 vol% and 85 vol% solutions of DES in ethanol were prepared. The viscosity of these solutions was measured at 5 temperature points—25 °C, 40 °C, 55 °C, 70 °C, and 85 °C, with the Modular Compact Rheometer (MCR 302, Anton Paar, Graz, Austria) with a titanium measuring cylinder.

### 2.3. Purification of the Spent DES

Purification of the spent DES was conducted by ultrafiltration (UF). The filtration experiments were performed in an Amicon dead end stirring cell unit (Millipore, Burlington, MA, USA, Cat No.: XFUF07611; the diameter of the stirring device 60 mm, while the effective filtration area was 40 cm^2^), at 45 °C, at 3.5 bar, at 250 rpm. The filterability of the solutions of spent DES and ethanol in different concentrations was studied. Filtration was performed in two ways—with a preliminary separation of the suspended solids and without it—with the second way (i.e., without preliminary separation) aiming at studying the effect of the presence of suspended solids on the performance and possible fouling of the membrane. The following spent solutions were filtered in this experiment: 40 vol%, 60 vol% and 80 vol% of spent DES in ethanol.

**Figure 1:**
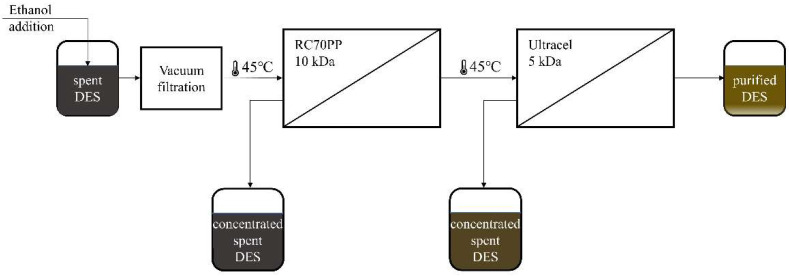
Spent DES purification process with multistage ultrafiltration.

Subsequently, 1 solution of spent DES in ethanol was chosen to be used in purification experiments of the spent DES. These experiments were performed in a multistage process (Figure 1), which was performed in the following way: the solution was pre-filtered through a 0.45 µm Nylon filter, then it was filtered through the RC70PP membrane in the Amicon cell unit. Thus, the RC70PP filtration was the first membrane filtration stage. After the retentate from the first stage had been collected, the permeate from the first stage was subsequently filtered through an Ultracel 5 kDa UF Disc in the Amicon cell unit. Retentates and permeates from the first and second stages were collected and further analyzed.

The pure water flux was measured before and after the filtration of the spent DES solution meaning that the DI water was filtered through a membrane for 10 min, at 21 °C, at 250 rpm and at each of the following pressures: 2 bar, 3 bar, and 4 bar. Membranes were compressed before measurement of the initial pure water fluxes by filtering pure water at 21 °C and at 250 rpm. The RC70PP membrane was compressed according to the procedure: 2 min at 2 bar, 4 min at 4 bar and 10 min at 6 bars. The procedure for the Ultracel 5 kDa UF Disc was different: 15 min at 3.5 bars.

### 2.4. Evaluation of the Resistance of the Cellulose Membranes to Fresh DES in Ethanol Solution

The RC70PP membranes were exposed to a 60 vol% solution of fresh DES in ethanol during the following periods of time: 1 week, 2 weeks, 3 weeks. Before the exposure to the DES, the pure water flux and retention values of the membranes were measured, according to the method described in Section 2.3. After the pure water flux measurements, polyethylene glycol (PEG) solution (300 ppm, 4 kDa) was filtrated through RC70PP at 2 bars, at 21 °C in the Amicon cell unit. The total carbon (TC) amount in retentates and permeates was analyzed on the TOC-L, Shimadzu Total Organic Carbon Analyzer and retention of the membranes was calculated based on these measurements.

### 2.5. Recovery of Lignin Fraction with Anti-Solvent Addition

Based on the results of the purification of the spent DES experiments, the 3 most feasible concentrations of spent DES in ethanol for filtration were identified (40 vol%, 50 vol% and 60 vol%). Solutions of these concentrations were firstly pre-filtered with vacuum filtration. Then, they were filtered through RC70PP at 21 °C and 45 °C separately. To analyze lignin from the filtration samples anti-solvent (deionized water 9 part and 1 part DES) addition was used to cause precipitation of lignin. After 5 min mixing of water and DES the solution was stored in a refrigerator for 16 h. Subsequently, the formed precipitates were separated from the solutions by vacuum filtration using a Buchner funnel, and the mass of the precipitate was measured.

### 2.6. Molecular Mass Analysis Using Size Exclusion Analysis (SEC)

A size exclusion chromatography analysis was conducted to obtain information on the molar mass distribution in the samples precipitated with anti-solvent from the DES fractions by using an Asahipak GS-320 HQ column with Asahipak GS-2G 7B guard column in 0.1 M NaOH (titrated with 35% HCl to pH 12.0) eluent with RI and UV detection. Results showed that the lignin concentration in the eluent was 0.5 g/L. The dimensions of the Asahipak GS-320 HQ column were as follows: 300 mm × 7.5 mm; manufacturer: Shodex, (Showa Denko America, Inc., New York, NY, USA), particle size: 6 μm; column temperature: 30 °C. In contrast, the dimensions of the Asahipak GS-2G 7B guard column were: 50 mm × 7.5 mm; manufacturer: Shodex, (Showa Denko America, Inc., New York, NY, USA), particle size: 9 μm; column temperature: 30 °C. Sodium polystyrene sulfonate molecular weight standards were used to determine the exclusion limit and the retention time calibration of the HPLC column.

### 2.7. Quantification of the Content of the Precipitated Fractions

The py-GC-MS was conducted to quantificate the content of the precipitated fractions on a foil pulse Pyrola 2000 pyrolyzer (Pyrol AB, Lund, Sweden) connected to the GC-MS instrument. The tentative identification was mainly based on the mass spectra library created at the Laboratory of Wood and Paper Chemistry at Åbo Akademi University and partly on the Wiley 10/NIST 2012 mass spectral libraries. A sample amount of about 100 μg and 1 drop of acetone was applied onto the Pt filament, the sample was allowed to dry, and the analysis was started 2 min after the filament was added into the probe [28].

## 3. Results and Discussion

### 3.1. Influence of the Concentration of DES on Filterability of the DES and Ethanol Solutions

**Figure 2:**
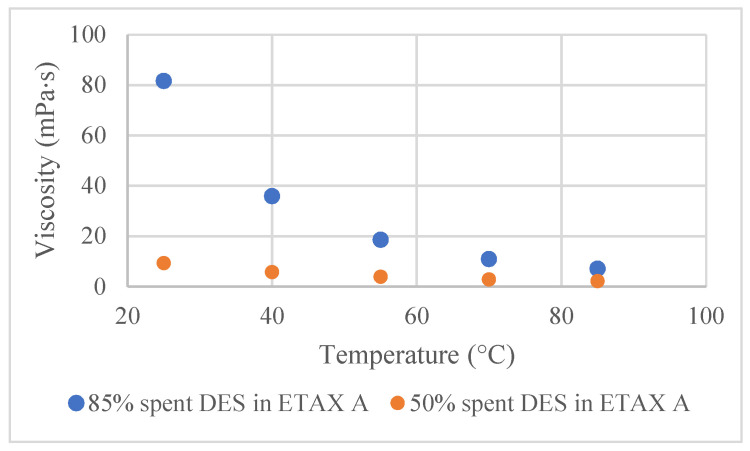
Influence of ethanol addition to the viscosity at different temperatures for the following spent DES in ethanol solutions: 85 vol% and 50 vol%.

Viscosity measurements showed that the viscosity values of DES in ethanol solutions of different concentrations were close to each other when the temperature was high (Figure 2). For example, at 25 °C, the difference in viscosity values between 85 vol% and 50 vol% solutions was about 70 mPa × s, while the difference between the same solutions at 85 °C was only 5 mPas × s. Hence, at 25 °C the cosolvent addition plays a significant role in DES’s viscosity reduction. With an increase in temperature, this role becomes less important. Even though the DES is diluted with ethanol, its viscosity is still clearly higher compared to the viscosity of water (around 1 mPas × s).

**Figure 3:**
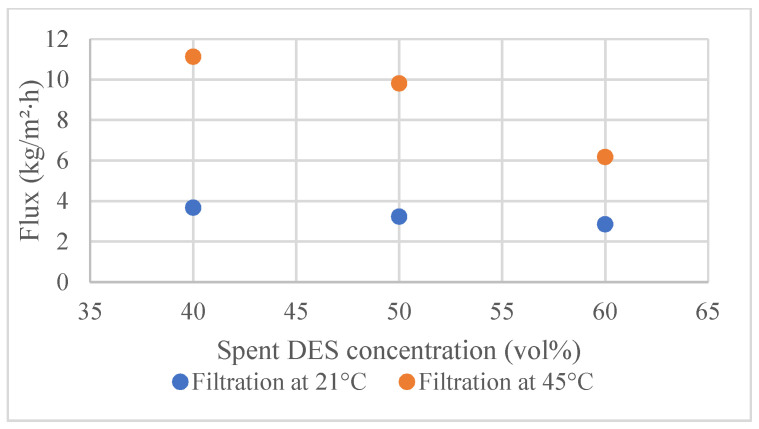
Effect of DES concentration in ethanol solution on the permeate flux through RC70PP at two temperatures—21 °C and 45 °C.

From the membrane point of view, the filtration temperature should not be very high: the manufacturer recommends the membranes to be used at temperatures between 5 and 60 °C (RC70PP) and between 5 and 50 °C (Ultracel 5 kDa UF Discs) [29,30]. Thus, with the cellulose membranes used here, the possibility of decreasing viscosity via an increase of filtration temperature is limited. However, the addition of cosolvent can enable the filtration through the decrease of viscosity at temperatures 21 °C and 45 °C, as can be seen in Figure 3. As was expected, flux decreased with the increase of the spent DES concentration at both tested temperatures. The right combination of the amount of cosolvent and filtration temperature can significantly decrease the viscosity of the spent DES without the need to operate at a very high temperature or solvent concentration.

As could be expected, the fluxes of 40 vol%, 50 vol%, and 60 vol% solutions of spent DES in ethanol at 45 °C were significantly higher than the fluxes of the same solutions at 21 °C. In fact, the flux of the spent DES solutions at 21 °C was so low that it was not reasonable to measure concentrations higher than 60 vol%. The results showed that the flux of 90 vol% solution at 45 °C was at the same level as the flux of 60 vol% solution at 21 °C.

The addition of cosolvent also caused some precipitation of polymeric compounds (mainly carbohydrates) in the spent DES. The Py-GC/MS analysis showed that carbohydrates precipitated predominantly over lignin: the amount of carbohydrates in the precipitation was 4.1 times bigger than the amount of lignin. The precipitation of carbohydrates prior to lignin is advantageous because it might enable the production of a lignin fraction with improved purity. Therefore, the removal of this precipitate prior to the membrane filtration is highly desirable also from the lignin purity point of view.

The filtration results revealed that removal of the precipitates (suspended solids) before the ultrafiltration significantly decreased the membrane’s loss of pure water permeability (Table 2). The loss of pure water permeability was always bigger for those solutions that were not pre-filtered prior to UF. The effect of suspended solids removal on the decrease in the loss of pure water permeability was greater for the higher spent DES solution concentration. This phenomenon stems from the fact that the higher amount of spent DES contains higher amounts of compounds, which are ready to precipitate with a cosolvent addition.

### 3.2. Stability of Cellulose Membrane in DES Solution Exposure

**Figure 4:**
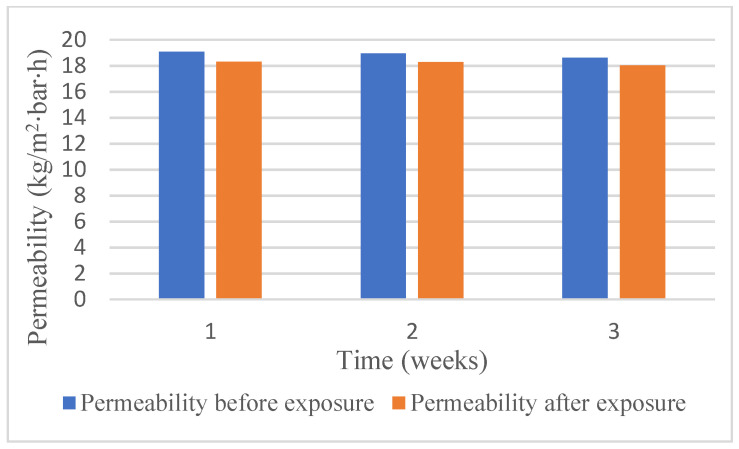
Permeabilities of RC70PP membranes exposed to 60 vol% fresh DES in ethanol for three time periods: 1 week, 2 weeks, 3 weeks.

The information on differences of pure water fluxes of the RC70PP membranes before and after the exposure of the membrane pieces to 60 vol% DES in ethanol is presented in Figure 4, and the effect of the exposure on the retention of the membrane samples is shown in Table 3.

It is noticeable from Figure 4 that permeability values before and after the exposure to the DES and ethanol were at the same level. According to Table 3, retention of RC70PP was not much influenced by the DES solution even after 3 weeks of exposure. Thus, summarizing the results from Figure 4 and Table 3, the RC70PP regenerated cellulose membrane seems to withstand the DES solution in ethanol for continuous periods of time without any significant deteriorations in its retention and pure water permeability performance.

### 3.3. Membranes’ Performance in Ultrafiltration

RC70PP and Ultracel 5 kDa membranes demonstrated low permeabilities in the conditions used on the multistage filtration process to purify the DES and recover lignin. The information on the permeabilities of these membranes is presented in Table 4. Due to the high viscosity of the spent DES solution, low fluxes were expected (Section 3.1). However, to enable a better filtration capacity, there is still a need for actions to decrease the viscosity and enhance the permeability. In addition to the changes in filtration conditions, a module enabling higher turbulence on the membrane surface might be a possibility.

Changes in pure water permeabilities of the membranes due to the DES are illustrated in Table 4. As can be seen in Table 4, the RC70PP was remarkably affected by the solution (41% of pure water permeability loss), while Ultracel 5 kDa seems to be affected very insignificantly (0.4% of pure water permeability loss). This result indicates that the loss of pure water permeability was not significant in the treatment of the RC70PP permeate with the cellulosic 5 kDa membrane. Such a result is promising, but to obtain a better idea on the fouling behavior of these membranes in the treatment of spent DES, a more detailed study is required.

### 3.4. Lignin Recovery with the Tested Ultrafiltration Membranes

**Figure 5:**
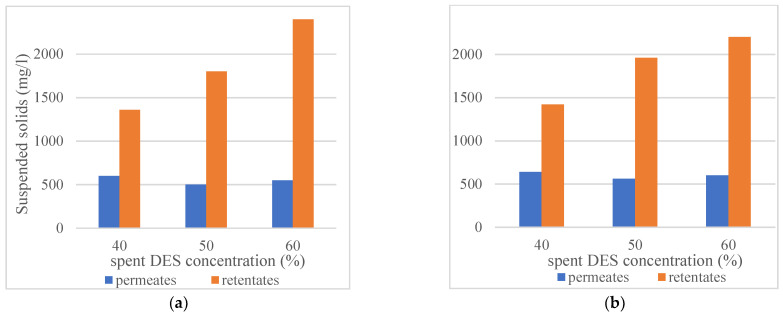
Suspended solids (hemicelluloses and lignin) in permeates and retentates of 40 vol%, 50 vol% and 60 vol% spent DES solutions after anti-solvent precipitation (RC70PP membrane, Amicon cell unit, at 3.5 bar, 21 °C and 45 °C, 250 rpm, anti-solvent: DES ratio 9:1): (**a**) Ultrafiltration at 21 °C; (**b**) Ultrafiltration at 45 °C.

Section 3.1 (Influence of the concentration of DES on filterability of the DES and ethanol solutions) established that the combination of cosolvent addition to spent DES and filtration at elevated temperature (45 °C) makes filtration more efficient. Figure 5 provides results on the amount of suspended solids after the precipitation with anti-solvent in permeates, and retentates of membrane filtrations with 40, 50, and 50% spent DES solutions. Filtration temperature has a negligible effect on the amounts of precipitated compounds, but obviously, the higher is DES concentration and, therefore, the amount of dissolved compounds in the solution, the higher amounts of compounds were precipitated by anti-solvent addition from the retentate samples. However, the DES concentration in ethanol solution did not have an influence on the anti-solvent-induced precipitation of compounds in the permeates from the 10 kDa membrane filtration.

Py-GC/MS analysis was performed to establish the presence of hemicellulose and lignin and their amounts in the precipitates of feed, permeate, and concentrate samples. Two-pass membrane filtration with 50% DES recovery in both filtration stages showed that the polymeric compounds (hemicellulose and lignin) could be very efficiently removed from the DES-ethanol solution. Retention of lignin, which was precipitated by anti-solvent addition with the 10 kDa membrane, was about 70% and with 5 kDa membrane about 95%.

**Figure 6:**
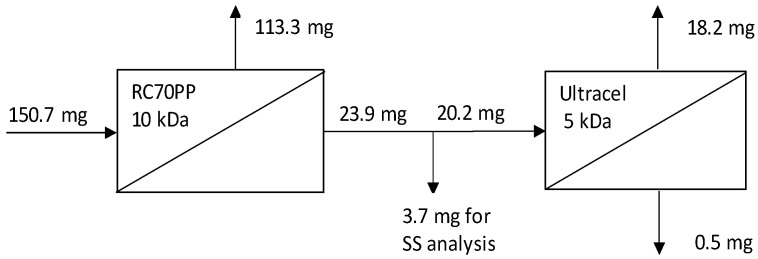
Mass balance of precipitated lignin (precipitation by water addition) for the two-pass membrane filtration process of 60 vol% spent DES in ethanol purification.

Based on Figure 6, it is evident that the use of the combination of RC70PP and Ultracel 5 kDa enabled lignin concentration in the retentate fractions produced in this multistage filtration. The lignin amount decreased from 150.7 mg in the feed for the first stage (RC70PP filtration) to 0.5 mg in the permeate from the last stage (Ultracel 5 kDa filtration), and most of the lignin precipitated by anti-solvent addition ended up in the retentate fraction of the RC70PP membrane (113.3 mg).

**Figure 7:**
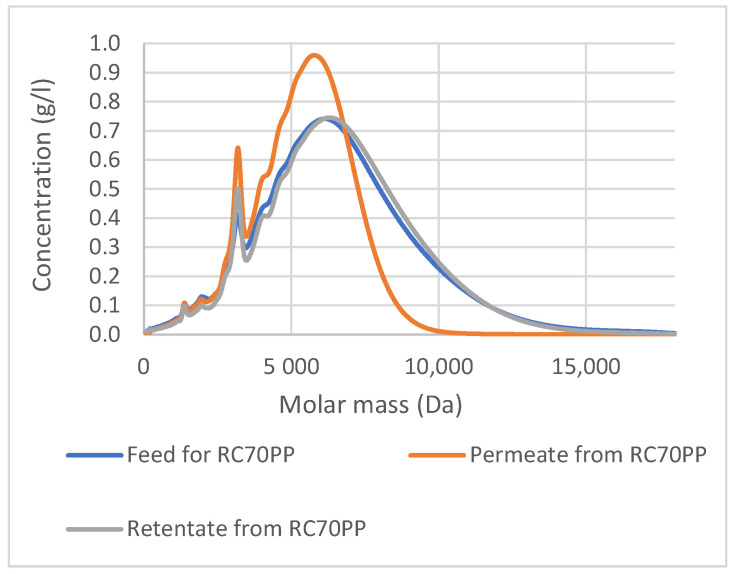
Correlation between molar mass distribution and concentration for the precipitates from the feed, the permeate and the retentate of RC70PP filtration, detected with a UV detector.

The retentate from RC70PP fraction contained molecules that have a higher molar mass than 10,000 Da, because, based on the SEC analysis (Figure 7), they were not permeating the RC70PP membrane. However, as can be seen from the molar mass distribution curves, the retentate fraction from the RC70PP also contained smaller molecules. In any case, the result shows the possibility to recover a higher molar mass fraction of the lignin from the spent DES with the RC70PP membrane. The fraction could, for instance, be washed in diafiltration if one wanted to remove the smaller molecules from the fraction.

The other fractions produced in the multistage filtration process contained only minor amounts of lignin compared to the RC70PP retentate. However, to purify the solvent from lignin before the evaporation of the ethanol to enable the recycling of the spent DES, there should be a further membrane step. As previously mentioned, the 5 kDa cellulose membrane enabled the removal of almost all the lignin, which could be precipitated from the spent DES with water.

## 4. Conclusions

A filtration process with regenerated cellulose membranes was applied in the purification of the spent DES used in the extraction of lignin from hardwood. Due to the very high viscosity of the spent DES solution, a cosolvent (ethanol) was added to enable membrane filtration. Filtration experiments were done with different spent DES in ethanol solutions at two temperatures. A 60% solution and a temperature of 45 °C were chosen for further experiments. The selected combination is a compromise of flux and the amount of the used cosolvent. Two-pass filtration process (RC70PP followed by the Ultracel 5 kDa) was investigated to purify DES solution. The cosolvent (ethanol) induced some precipitation. However, Py-GC/MS analysis of the precipitate demonstrated that it contained more carbohydrates than lignin.

Anti-solvent induced precipitation of polymeric compounds, and Py-GC/MS analysis of precipitates was used to evaluate the efficiency of membrane filtration to purify the DES solution from polymeric compounds (lignin and hemicelluloses). Two-pass membrane filtration with 50% DES recovery in both filtration stages showed that the polymeric compounds (hemicellulose and lignin) could be very efficiently removed from the DES-ethanol solution. Retention of the compounds, which were precipitated by anti-solvent addition with the 10 kDa membrane, was about 70% and with 5 kDa membrane about 95%. The purified parts of spent DES (permeates) can be directly recycled after ethanol evaporation, while the concentrated parts of spent DES can be used in lignin recovery with minimized, compared to untreated spent DES, water consumption. This study also showed that the RC70PP membrane withstood the three weeks of exposure to the solution of ethanol and spent DES very well. Thus, the results of this study are promising as they show the possibility to use cellulosic ultrafiltration membranes in the process, enabling both the recovery of a high molar mass lignin fraction and recycling of the spent DES. The process still needs development, for instance, to enable higher filtration capacity and minimize the utilization of fresh solvents. These research questions will be investigated in further research.

## Figures and Tables

**Table 1:** Technical characteristics of the RC70PP membrane (Alfa Laval company) and the Ultracel UF Discs 5 kDa (Millipore company).

Membrane Type	Material	MWCO Value, Da	pH Range	Maximum Operating Pressure, Bar	Maximum Operating Temperature, °C
RC70PP	Regenerated cellulose acetate	10,000	1–10	10	60
Ultracel 5 kDa	Regenerated cellulose	5000	3–13	4.8	50

**Table 2:** Permeability of RC70PP before and after filtration. Filtration parameters: 3.5 bar, 45 °C, 250 rpm. The solutions without suspended solids (“Absent”) had a prior Buchner filtration step before UF filtration.

Initial Suspended Solids	Spent DES Concentration, vol%	Pure Water Permeability before, kg/m^2^⋅h	Pure Water Permeability after, kg/m^2^⋅h	Loss of Pure Water Permeability, %
Present	40	19.3	10.4	46
	60	16.2	8.3	49
	80	18.8	7.1	63
Absent	40	15.6	12.3	21%
	60	16.7	14.7	12%
	80	18.8	15.3	14%

**Table 3:** Total carbon retentions of the membranes exposed to 60 vol% fresh DES in ethanol measured with PEG (4 kDa) solution of 300 ppm.

Time Period	Retention Before Exposure, %	Retention After Exposure, %
1 week	95.2	86.6
2 weeks	94.2	94.1
3 weeks	93.2	95.1

**Table 4:** Permeability of the spent DES solution filtration and pure water permeabilities before and after UF through RC70PP and Ultracel 5 kDa, Amicon cell unit. Parameters of the spent DES solution filtration: 3.5 bar, 45 °C, 250 rpm. Parameters of the pure water filtration: 10 min at 2 bar, 3 bar, 4 bar, 21 °C, 250 rpm.

Membrane Type	Permeability for the Spent DES Solution Filtration, kg/m^2^⋅h⋅bar	Pure Water Permeability before, kg/m^2^⋅h⋅bar	Pure Water Permeability after, kg/m^2^⋅h⋅bar	Loss of Pure Water Permeability, %
RC70PP	1.15	14.76	8.72	40.9
Ultracel 5 kDa	0.27	4.90	4.88	0.4

## Data Availability

The data presented in this study are available on request from the corresponding author.
